# Fibroblast Growth Factor-4 Enhances Proliferation of Mouse Embryonic Stem Cells via Activation of c-Jun Signaling

**DOI:** 10.1371/journal.pone.0071641

**Published:** 2013-08-13

**Authors:** Sung-Ho Kook, Young-Mi Jeon, Shin-Saeng Lim, Moon-Ju Jang, Eui-Sic Cho, Seung-Yeop Lee, Ki-Choon Choi, Jong-Ghee Kim, Jeong-Chae Lee

**Affiliations:** 1 Cluster for Craniofacial Development and Regeneration Research, Institute of Oral Biosciences and School of Dentistry, Chonbuk National University, Jeonju, South Korea; 2 Research Center of Bioactive Materials, Chonbuk National University, Jeonju, South Korea; 3 Grassland and Forages Research Center, National Institute of Animal Science, Cheonan, South Korea; University of South Alabama Mitchell Cancer Institute, United States of America

## Abstract

Fibroblast growth factor-4 (FGF4) is expressed in embryonic stages and in adult tissues, where it plays critical roles in modulating multiple cellular functions. However, the exact roles of FGF4 on proliferation and differentiation of embryonic stem cells (ESCs) are not completely understood. Exogenous addition of FGF4 stimulated proliferation of mouse ESCs (mESCs), as proven by the increases in DNA synthesis and cell cycle regulatory protein induction. These increases were almost completely inhibited by pre-treating cells with anti-FGF4 antibody. FGF4 also activated c-Jun N-terminal kinase (JNK) and extracellular-signal regulated kinase (ERK) signaling, but not p38 kinase. Blockage of JNK signaling by SP600125 or by transfection with its specific siRNA significantly inhibited FGF4-stimulated cell proliferation through the suppression of c-Jun induction and activator protein-1 (AP-1) activity. However, ERK or p38 kinase inhibitor did not affect FGF4-stimulated proliferation in mESCs. FGF4 suppressed osteogenic differentiation of mESCs by inhibiting expression of transcription factors involved in bone formation. Further, exogenous FGF4 addition stimulated proliferation of human periodontal ligament stem cells (hPDLSCs) and bone marrow mesenchymal stem cells (BMMSCs) via activation of ERK signaling. FGF4 also augmented mineralization of hPDLSCs, but not of BMMSCs. Collectively, it is suggested that FGF4 triggers proliferation of stem cells by activating MAPK-mediated signaling, while it affects differently osteogenic differentiation according to the origins of stem cells.

## Introduction

Fibroblast growth factor (FGF) plays essential roles in multiple biological processes including cellular proliferation, differentiation, and survival [1], [2]. Approximately 24 members of the FGF family have been identified and their functions differ according to the FGF family and cell type from which they were derived. According to the previous reports [3]–[5], the ability of FGF family to modulate cellular functions depends on the type and origin of cells examined.

FGF4 is the first FGF detected during embryonic development. This factor is an autocrine and/or paracrine growth factor required for multiple cellular events during embryogenesis [6]. It was previously found that FGF4 increases proliferation of neural progenitors [7] or bone marrow mesenchymal stem cells (BMMSCs) [8] and sustains the survival of trophoblast stem cells [9]. These findings indicate that FGF4 plays a predominant role in stimulating cell proliferation. However, other studies have shown that exogenous FGF4 addition did not increase proliferation of embryonic stem cells (ESCs) [10], [11]. This suggests that FGF4 may have different roles depending on the developmental stages of stem cells and their origin. It is also still unclear whether FGF4 is an essential growth factor for proliferation of ESCs, even though FGF4 has been shown to control stem cell fate and proliferation of many types of cells. The molecular mechanisms by which FGF4 regulates proliferation and differentiation of ESCs are not entirely defined.

Mitogen-activated protein kinases (MAPKs) are major signal mediators in response to various stimuli such as growth factors, cytokines, and stress [12]–[14]. There are three kinds of MAPKs including c-Jun N-terminal protein kinase (JNK), extracellular signal-regulated kinase (ERK), and p38 kinase. These kinases are critical for regulating proliferation and differentiation of stem cells in response to FGFs [15], [16]. It is commonly accepted that FGFs exert their effects by activating receptor tyrosine kinases of the FGF receptor family, which eventually leads to activation of Ras-Raf-MAPK signaling pathways [17]. For example, addition of FGF2 stimulates proliferation of mesenchymal stem cells (MSCs) through activation of JNK-mediated signaling [4]. Exogenous FGF2 and 4 enhanced proliferation of bone marrow MSCs (BMMSCs) by activating PI3K-Akt and ERK1/2 signaling [8], [18]. These previous reports proposed that FGF4 may play its predominant role in stimulating proliferation and differentiation of ESCs via MAPK-mediated signaling pathways.

In this study, we examined the effects of exogenous FGF4 on proliferation and osteogenic differentiation of mouse ESCs (mESCs). We also investigated the cellular mechanisms by which FGF4 affects proliferation and osteoblastic differentiation of mESCs. In addition, we investigated the effects of FGF4 on human periodontal ligament stem cells (hPDLSCs) and mouse BMMSCs (mBMMSCs). Our present findings show that exogenous FGF4 addition stimulates proliferation of mESCs as well as hPDLSCs and mBMMSCs via the activation of MAPK-mediated signaling. In contrast, FGF4 exerts different roles on osteogenic differentiation according to the origins of stem cells, where it inhibits osteoblastogenesis of mESCs, but accelerates mineralization of hPDLSCs.

## Materials and Methods

### Chemicals and Laboratory Wares

The mouse ESC line D3 was obtained from the American Type Culture Collection (Rockwille, MD, USA) and fetal bovine serum (FBS) was purchased from Gibco-BRL (Gaithersburg, MD, USA). MAPK inhibitors including SP600125, PD98059, and SB203580 were purchased from TOCRIS (Bristol, UK) and dissolved in absolute ethanol or DMSO prior to use. All antibodies specific for MAPKs, cell cycle regulatory factors, and cell surface markers were obtained from Santa Cruz Biotechnology Inc. (Santa Cruz, CA, USA). Unless specified otherwise, other chemicals and laboratory wares were obtained from Sigma Chemical Co. (St. Louis, MO, USA) and SPL Life Sciences (Pochun, South Korea), respectively.

### Cell Culture and FGF4 Treatment

mESCs were cultured in Dulbecco’s modified Eagle’s medium (DMEM) (Gibco-BRL, Gaithersburg, MD, USA) supplemented with 1.7 mM L-glutamine, 0.1 mM β-mercaptoethanol, 5 ng/ml mouse leukemia inhibitory factor (LIF), 15% FBS, and 1% penicillin and streptomycin, without a feeder layer at 37°C in an atmosphere containing air and 5% CO_2_. For the cell proliferation assay, human recombinant FGF4 (R&D Systems, Inc., Minneapolis, MN, USA) was added with various concentrations into serum-free DMEM and further incubated for various times in the presence of anti-human FGF4 antibody (R&D System, Inc.) or each inhibitor of MAPKs. hPDLSCs were isolated from healthy human molars according to guidelines approved by the Review Board of Chonbuk National University [19]. These cells were cultured in α-MEM containing 10% FBS, penicillin (100 U/ml), and streptomycin (100 µg/ml), as described previously [20]. mBMMSCs were isolated from 6 to 8 week-old ICR mice by flushing the femurs according to the methods previously described [8]. All animal produces were performed in accordance with guidelines approved by the Chonbuk National University Committee on Ethics in the Care and Use of Laboratory Animals (CBU 2010–0007). After 2 passages, hPDLSCs and mBMMSCs were subjected to magnetic isolation with autoMACS (Miltenyi Biotec, Cambridge, MA, USA) and the resulting cell populations were used for further studies after analyzing stem cell surface markers using flow cytometer (CyFlow® SL, Partec, Münster, Germany). All the experiments were carried out with passage 4–6 cells.

### Phenotypic Analysis of hPDLSCs and mBMMSCs

These cells were treated with 0.25% trypsin/EDTA and then resuspended in PBS supplemented with 2% FBS at passage 3. After fixation with 4% paraformaldehyde for 5 min, hPDLSCs were incubated with CD45, CD90, CD105, and CD146, while mBMMSCs were with antibodies including CD29, CD44, CD106, and Sca-1 for 20 min. These cells were washed and analyzed by flow cytometry (CyFlow® SL) after exposing to FITC-conjugated secondary antibody. Negative controls were stained with secondary antibodies only.

### BrdU Incorporation Assay

BrdU incorporation into DNA was detected using a BrdU incorporation assay kit according to the manufacturer’s instructions (Roche Molecular Biochemicals, Penzberg, Germany). Briefly, mESCs cultured in the presence of 0 to 200 ng/ml FGF4 for 48 h were exposed to 10 µM of BrdU for 4 h. Cells were incubated with anti-BrdU-peroxidase conjugate for 90 min before adding a tetramethylbenzidine substrate. Finally, reactive products were quantified by measuring absorbance at 450 nm with an ELISA reader (Packard Instrument Co., Downers Grove, IL, USA).

### Hematoxylin Staining

mESCs exposed to 50 ng/ml FGF4 for 48 h were fixed with 10% formalin for 30 min and stained with a hematoxylin solution (51275, Fluka Chemie GmbH, Steinheim, Switzerland) at room temperature for 5 min. Stained cells were photographed using a Nikon E5400 digital camera mounted on an optical microscope (Nikon TS100, Nikon Corporation, Japan).

### Tritium Uptake Assay

Rate of DNA synthesis in mESCs exposed to 50 ng/ml FGF4 and/or 100 ng/ml anti-FGF4 antibody for 48 h was determined by adding 0.5 µCi of [*methyl*-^3^H] thymidine deoxyribose (TdR; Amersham Pharmacia Biotech Inc., Piscataway, NJ, USA) to each well of 96-multiwell plates for an additional 16 h. After incubation, cells were collected using a cell harvester (Inotech Inc., Switzerland). Beta emission from ^3^H-TdR-incorporated cells was measured for 1 min using a liquid scintillation counter (Packard Instrument Co.).

### Cell Proliferation Assay

Proliferation rates of mESCs, hPDLSCs, and mBMMSCs were measured using the Cell Counting Kit-8 (Dojindo Laboratories, Kumamoto, Japan) according to the manufacturer’s instructions. In brief, these cells cultured in 96-multiwell plates were exposed to 50 ng/ml FGF4 and/or 100 ng/ml anti-FGF4 antibody for 48 h and then treated with 10 µl/well of the kit solution. Absorbance was measured at 450 nm using a microplate reader (Packard Instrument Co.).

### Western Blot Analysis

mESCs were cultured in the presence of 50 ng/ml FGF4 and/or 100 ng/ml anti-FGF antibody in 6-well culture plates for various times (0–24 h). These cells were lysed in NP-40 lysis buffer (30 mM Tris-Cl, pH 7.5, 1 mM EDTA, 150 mM NaCl, and 1% NP-40) and protein contents were quantified using the Bradford method [21]. Equal amounts of protein extracts were separated by 12–15% SDS-PAGE and blotted onto poly vinyl difluoride membranes. Blots were probed with primary antibodies overnight at 4°C prior to incubation with secondary antibody in blocking buffer for 1 h. Blots were developed with enhanced chemiluminescence (Amersham Pharmacia Biotech Inc., Buckinghamshire, UK) and exposed to X-ray film (Eastman-Kodak, Rochester, NY, USA). All antibodies (p-ERK; p-JNK; p-p38; cyclin D_1_, A, and B; proliferating cell nuclear antigen (PCNA); c-Jun; and p-c-Jun) were obtained from Santa Cruz Biotechnology Inc. (Santa Cruz, CA, USA). α-Tubulin antibody was purchased from Sigma Chemical Co. Primary antibodies and their dilution factors used in this study were as follows: p-JNK (1∶200), p-ERK1/2 (1∶200), p-p38 (1∶200), cyclin D_1_ (1∶200), cyclin A (1∶200), cyclin B (1∶100), PCNA (1∶200), p-c-Jun (1∶200), c-Jun (1∶200), and α-tubulin (1∶1,000).

### Immunostaining and Flow Cytometric Analysis

Levels of PCNA and runt-related transcription factor 2 (Runx2) in mESCs were measured by flow cytometry. For PCNA determination, cells were exposed to 50 ng/ml FGF4 and/or 100 ng/ml anti-FGF4 antibody for 24 h and then fixed in 4% paraformaldehyde for 15 min at room temperature. In addition, mESCs incubated in osteogenic differentiation medium for 3 days were fixed for analysis of Runx2. After washing, approximately 10^6^ cells were resuspended in PBS containing 0.1% BSA and incubated with primary antibody specific for PCNA or Runx2 for 30 min before exposure to goat anti-rabbit IgG-fluorescein isothiocyanate (FITC; Santa Cruz Biotechnology). Samples were analyzed using a flow cytometer (CyFlow^®^ SL) and approximately 10,000 events were recorded for each sample.

### MAPK Activity Assay

MAPK activity was determined using immunometric assay kits according to the manufacturer’s instructions. In brief, mESCs were exposed to 50 ng/ml FGF4 and/or 100 ng/ml anti-FGF4 antibody for 1 h and then cell lysates were prepared using RIPA buffer (50 mM Tris-Cl, pH 7.4, 150 mM NaCl, 1 mM EDTA, 1 mM EGTA, 1% Triton X-100, 1% sodium deoxycholate, and 0.1% SDS). After quantifying protein content using the Bradford method, equal amounts of protein samples were placed into microtiter plates of the p-p38 kinase assay kit (Assay Designs, Inc., Ann Arbor, MI, USA), p-ERK enzyme assay kit (Assay Designs), or p-SAPK/JNK sandwich ELISA kit (Cell Signaling Technology, Beverly, MA, USA). Absorbance was measured with a microplate reader.

### Small Interfering RNA Transfection

RNA interference of JNK was performed using 19-bp siRNA duplexes purchased from Ambion (Austin, TX, USA). The coding strand for JNK siRNA was 5′-TGA AAG AAT GTC CTA CCT T-3′ [22]. A non-related control siRNA that targeted the green fluorescent protein (GFP) sequence 5′-CCA CTA CCT GAG CAC CCA GTT-3′ was used as a control. For transfection, mESCs were seeded in 6-well plates and transfected with siRNA duplexes using Lipofectamine 2000 (Invitrogen) according to the manufacturer’s instructions. At 24 h post-transfection, mESCs were exposed to 50 ng/ml FGF4 and then processed for proliferation analysis, Western blotting, and electronic mobility shift assay (EMSA).

### DNA-Protein Binding Assay

Nuclear proteins from mESCs exposed to 50 ng/ml FGF4 with or without each inhibitor of MAPKs for 2 h were prepared as described previously [23] and quantified using the Bradford method. DNA-protein binding reactions by EMSA were carried out for 30 min at room temperature with 10–15 µg of protein in a 20-µl volume containing 1 µg/ml BSA, 0.5 µg/µl poly (dI-dC), 5% glycerol, 5 mM MgCl_2_, 1 mM DTT, 1 mM PMSF, 10 mM Tris-Cl (pH 7.5), 50 mM NaCl, 30,000 cpm of [α-^32^P] dCTP-labeled oligonucleotides, and the Klenow fragment of DNA polymerase. Samples were separated on 6% polyacrylamide gels, dried, and then exposed to X-ray films (Eastman Kodak Co., Rochester, NY, USA) for 12–48 h at −70°C. The following oligonucleotide primer sequences were used for EMSA: 5′-AAGGGATCCGGCTGACTCATCACTAG-3′ and 3′-CTAGGCCGACTGAGTAGTGATCGGAA-5′ for activator protein (AP)-1.

### AP-1 Activity Assay

When mESCs reached 80–90% confluence, cells were incubated in serum-free culture medium for 24 h to minimize basal AP-1 activity. Cells were exposed to 50 ng/ml FGF4 for 2 h, and luciferase activity specific to AP-1 was then measured using a luciferase assay kit (Promega, Madison, WI, USA) as described elsewhere [24]. Results are expressed as relative AP-1 activity compared with controls.

### Osteogenic Differentiation

To induce osteoblastic differentiation of mESCs, hPDLSCs, and mBMMSCs, these cells were treated with osteogenic medium (10% FBS, 100 nM dexamethasone, 50 µM ascorbic acid, and 5 mM β-glycerophosphate in α-MEM) in the presence or absence of 50 ng/ml FGF4. At various times (0–14 days), cells were processed for analysis of proliferation, mineralization, alkaline phosphatase (ALP) activity, and expression of bone-specific genes.

### Alizarin Red Staining

The degree of mineralization in cells was determined in 6-well plates using Alizarin red staining (Sigma Chemical Co). In brief, three types of stem cells were incubated in osteogenic medium for various times and then fixed with ice-cold 70% (vol/vol) ethanol for 1 h. Cells were stained with 0.2% Alizarin red S in distilled water for 30 min at room temperature. After destaining and air-drying, cell culture plates were evaluated by light microscopy using an inverted microscope (Nikon TS100). In order to quantify the red dye, stains were also solubilized with 10% cetylpyridinum chloride by shaking for 20 min and absorbance was measured at 560 nm.

### Measurement of ALP Activity

ALP activity was determined by a biochemical colorimetric assay using the Sigma-Aldrich Alkaline Phosphatase assay kit (104-LS) according to manufacturer’s instructions. In brief, three types of stem cells were incubated in osteogenic medium with or without FGF4 and anti-FGF4 for 3 days. Cells were washed with PBS and then sonicated in PBS containing 0.01% SDS to prepare cell lysates. Enzymatic reaction was started after mixing cell lysates with an enzymatic reaction buffer containing *p*-nitrophenyl phosphate substrate. The reaction was stopped by adding 1 ml of 0.05 N NaOH and absorbance was read at 405 nm using an ELISA reader. Finally, ALP activity was calculated from a standard curve after normalization of protein levels using the Bradford method.

### Real-Time Reverse Transcriptase-Polymerase Chain Reaction (RT-PCR)

Total RNA was extracted with Trizol reagent (Invitrogen Corp., Carlsbad, CA, USA). Total RNA (1 µg) was subjected to cDNA synthesis with SuperScript Reverse Transcriptase II and oligo_12–18_ primers (Invitrogen). Power SYBR Green PCR Master Mix (Applied Biosystems, Foster City, CA, USA) was used to detect accumulation of PCR product during cycling with the ABI 7500 sequence detection system (Applied Biosystems). Thermocycling conditions were as follows: initial denaturation at 95°C for 10 min followed by 40 cycles of denaturation at 95°C for 15 sec, annealing at 60°C for 60 sec, and extension at 72°C for 30 sec. Positive standards and reaction mixtures lacking reverse transcriptase were used routinely as controls for each RNA sample. Oligonucleotide primers were designed with product sizes less than 200 bp using Primer Express 3.0 (Applied Biosystems) as shown in Table 1.

**Table 1:** The primer sequences used to analyze the expression of bone-specific genes.

Gene name	Gene bank	sequences		product size (bp)
GAPDH	NM_008084.2	F	GACGGCCGCATCTTCTTGT	65
		R	CACACCGACCTTCACCATTTT	
Runx2	NM_009820.4	F	GAGGGACTATGGCGTCAAACA	70
		R	GGATCCCAAAAGAAGCTTTGC	
Osterix	NM_130458.2	F	TCAGCCGCCCCGATCTTCCA	156
		R	AATGGGTCCACCGCGCCAAG	
OPN	NM_009263.1	F	TGGTGGTGATCTAGTGGTGCCAA	148
		R	CACCGGGAGGGAGGAGGCAA	
ALP	NM_007431.2	F	GGGCCCTGCTGCTTCCACTG	70
		R	GGCTTGTGGGACCTGCACCC	
OC	NM_001037939.1	F	ACTCCGGCGCTACCTTGGGT	109
		R	CCTGCACGTCTAGCCCTCTGC	
BSP	NM_008318.2	F	AGACCAGGAGGCGGAGGCAG	123
		R	TTGGGCAGTTGGAGTGCCGC	

### Statistical Analysis

Unless specified otherwise, all data are expressed as the mean ± standard deviation (SD). One-way analysis of variance (ANOVA, SPSS version 16.0 software) followed by Scheffe’s test were applied to determine significant differences between groups. A p value<0.05 was considered statistically significant.

## Results

### FGF4 Stimulates Proliferation of mESCs

**Figure 1:**
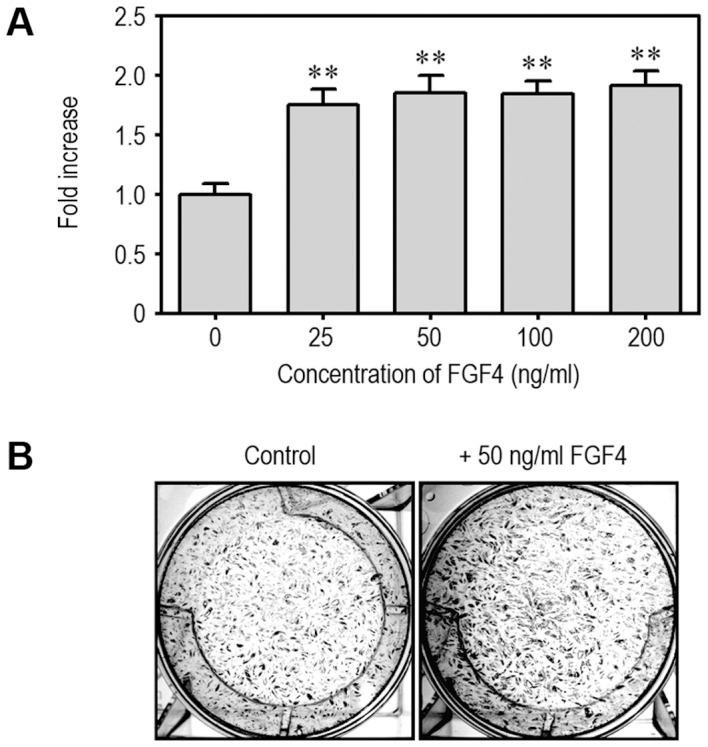
FGF4 increases proliferation of mESCs. mESCs were incubated in the presence of 0 to 200 ng/ml FGF4 in 96-multiwell plates or with the indicated concentration of FGF4 in 6-well culture plates for 48 h and then further processed for BrdU incorporation assay (A) and hematoxylin staining (B), respectively. Experimental results shown in (A) represent the mean ± SD from three separate experiments. **p<0.01 indicates a significant difference between experiments and untreated control cells.

The effect of FGF4 on proliferation of mESCs was determined using an immunoassay based on the measurement of BrdU incorporation at 48 h after treatment. BrdU incorporation was increased up to approximately 1.75-fold in cells treated with 25 ng/ml FGF4 for 48 compared with control cells (Fig. 1A). Proliferation of mESCs reached a maximum at 200 ng/ml FGF4, whereas no significant differences between FGF4 concentrations ranging from 25 to 200 ng/ml were observed. When cells were stained with hematoxylin at the same time, the numbers of positively stained cells were also higher in FGF4-treated group than non-treated population (Fig. 1B). These results suggest that FGF4 stimulates proliferation of mESCs.

### Anti-FGF4 Antibody Inhibits FGF4-Stimulated Proliferation and Cell Cycle Regulatory Protein Induction

**Figure 2:**
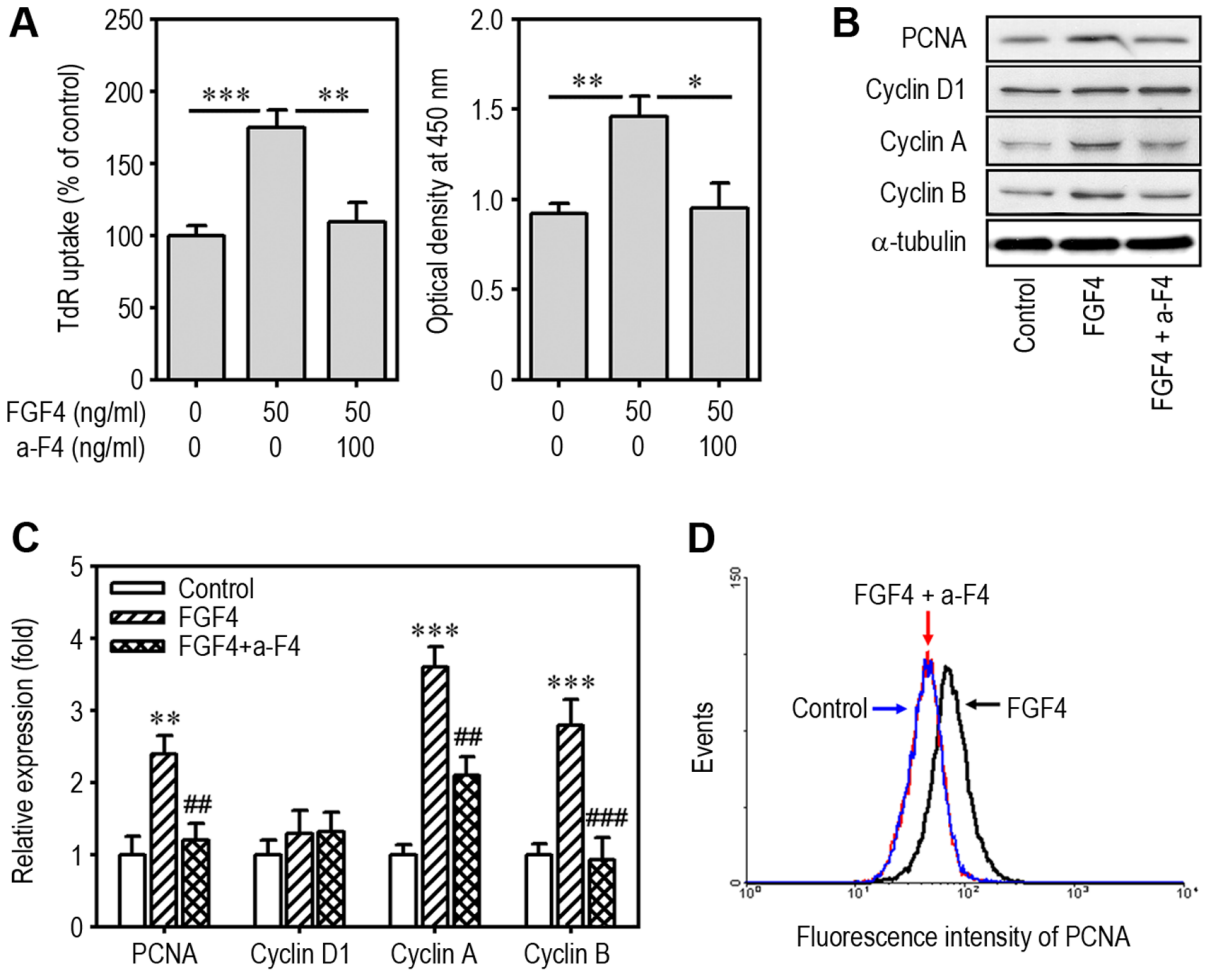
Effects of anti-FGF4 antibody on FGF4-induced cell proliferation and protein expression associated with cell cycle progression. mESCs cultured in 6-well culture plates were incubated in the presence of 50 ng/ml FGF4 and/or 100 ng/ml anti-FGF4 antibody and then proliferation rates were analyzed using ^3^H-TdR incorporation (A, left panel) and Cell Counting Kit-8 (A, right panel) 48 h after treatment. *p<0.05, **p<0.01, and ***p<0.001 indicate significant differences between experiments. (B) Expression patterns of cell cycle regulatory proteins 24 h after treatment were analyzed by Western blotting. (C) Data from Western blot analysis were quantified by densitometry after normalizing bands to α-tubulin levels. **p<0.01 and ***p<0.001 vs. untreated cells. ^##^p<0.01 and ^###^p<0.001 vs. cells treated with FGF4 only. (D) mESCs cultured under the same conditions as (C) were also processed for analysis of PCNA expression by flow cytometry. a-F4, anti-FGF4 antibody.

In order to clarify the stimulating effect of FGF4 on cell proliferation, mESCs were incubated in the presence of 50 ng/ml FGF4 with or without 100 ng/ml anti-FGF4 antibody. Levels of ^3^H-TdR incorporated by cells (Fig. 2A, left panel) and cell proliferation measured with the Cell Counting Kit-8 (Fig. 2A, right panel) were increased by exogenous addition of FGF4. These increases were significantly suppressed by treatment with anti-FGF4 antibody. The BrdU incorporation assay also supported FGF4-stimulated proliferation and its inhibition by anti-FGF4 antibody (data not shown). Western blot analysis showed that FGF4 enhanced cellular levels of PCNA, cyclin A, and cyclin B proteins, whereas the addition of anti-FGF4 antibody reduced them to basal levels (Figs. 2B and C). In addition, FGF4-mediated increase in fluorescence intensity specific for PCNA was reduced to level similar to that of non-treated cells (Fig. 2D).

### FGF4 Increases Phosphorylated Levels of JNK and ERK, but not p38 Kinase, in mESCs

**Figure 3:**
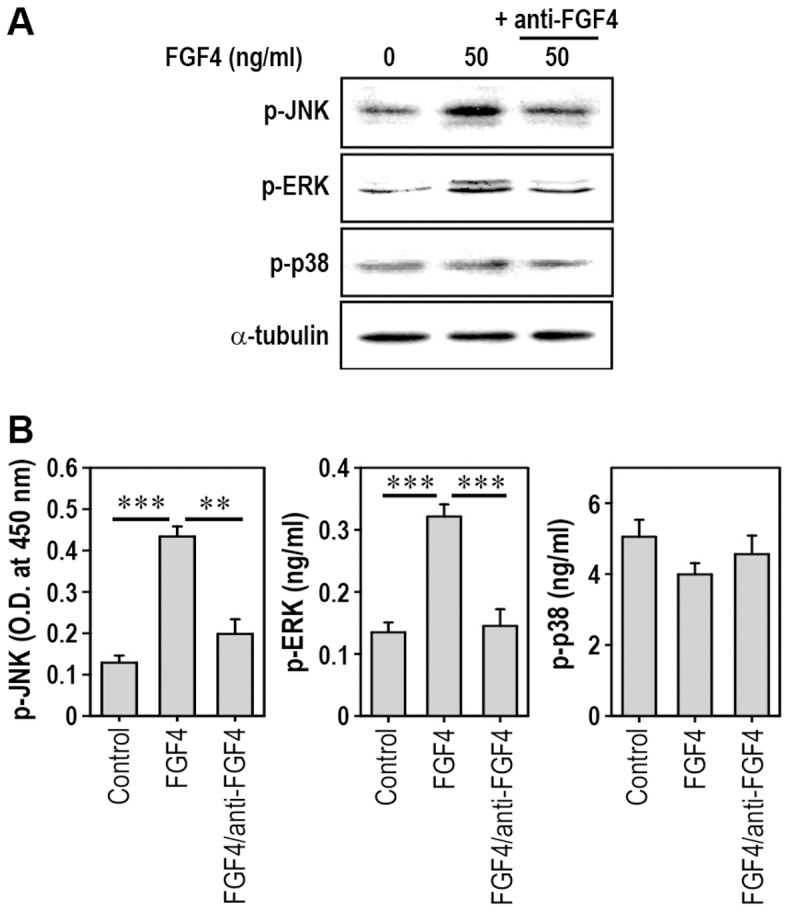
Addition of FGF4 activates JNK and ERK, but not p38 kinase, in mESCs. mESCs cultured in 6-well culture plates were incubated in the presence of 50 ng/ml FGF4 and/or 100 ng/ml anti-FGF4 antibody for 1 h and then processed to determine levels of phosphorylated MAPK by immunoblotting (A) or by immunometric analyses (B). **p<0.01 and ***p<0.001 indicate significant differences between experiments.

We examined levels of phosphorylated MAPKs in mESCs. Total protein levels of JNK, ERK, or p38 kinase remained unchanged in FGF4-treated cells, regardless of the presence of its specific antibody (data not shown). Levels of p-JNK and p-ERK were clearly increased at 1 h after FGF4 treatment, whereas this increase was prevented by treatment with anti-FGF4 antibody (Fig. 3A). However, there was no change in p-p38 level in FGF4-treated cells, regardless of the addition of its antibody. Consistent with these results, immunometric assays also showed FGF4-stimulated increases in p-JNK and p-ERK levels and their suppression by anti-FGF4 antibody (Fig. 3B).

### FGF4 Stimulates Proliferation of mESCs through Activation of JNK Signaling

**Figure 4:**
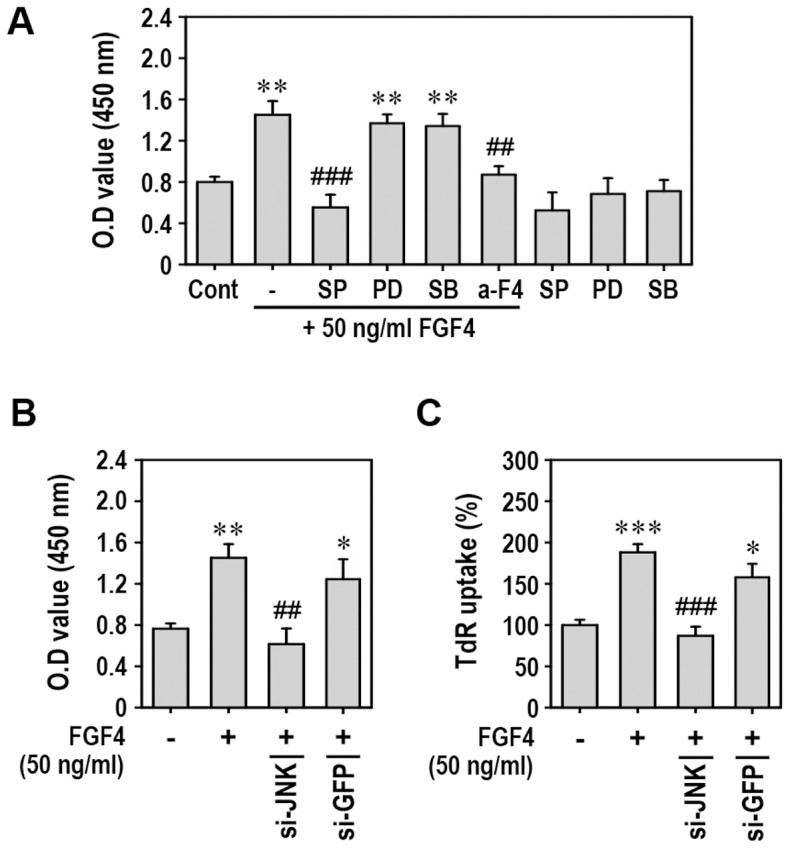
Inhibition of JNK suppresses FGF4-mediated proliferation of mESCs. (A) mESCs were pretreated with MAPK inhibitor or anti-FGF4 antibody 30 min before addition of 50 ng/ml recombinant FGF4, and cell proliferation rate was determined 48 h after treatment using a cell proliferation assay kit. In addition, siRNAs directed against JNK or GFP were transfected into mESCs. At 24 h post-transfection, cells were exposed to 50 ng/ml FGF4 and cell proliferation rate (B) and ^3^H-TdR incorporation (C) were measured 48 h after treatment. *p<0.05, **p<0.01, and ***p<0.001 vs. untreated cells. ^##^p<0.01 and ^###^p<0.001 vs. cells treated with FGF4 only.

To further examine the roles of MAPKs on FGF4-stimulated proliferation, mESCs were exposed to 50 ng/ml FGF4 in the presence of each inhibitor specific to MAPKs. Pretreatment with SP600125, but not with PD98059 or SB203580, completely prevented FGF4-stimulated proliferation of the cells (Fig. 4A). MAPK inhibitor itself did not change the rate of proliferation of the cells. Consistent with this result, the blockage of JNK signaling by transfection with its specific siRNA significantly inhibited FGF4-mediated increases in the number of cells (Fig. 4B) and the levels of DNA synthesis (Fig. 4C). On the other hand, siRNA transfection specific for ERK or p38 kinase did not affect FGF4-stimulated proliferation of mESCs (data not shown). These findings suggest that FGF4 stimulates proliferation of mESCs by activating JNK signaling.

### c-JUN and AP-1 are closely Associated with JNK-Mediated Proliferation in FGF4-Stimulated Cells

**Figure 5:**
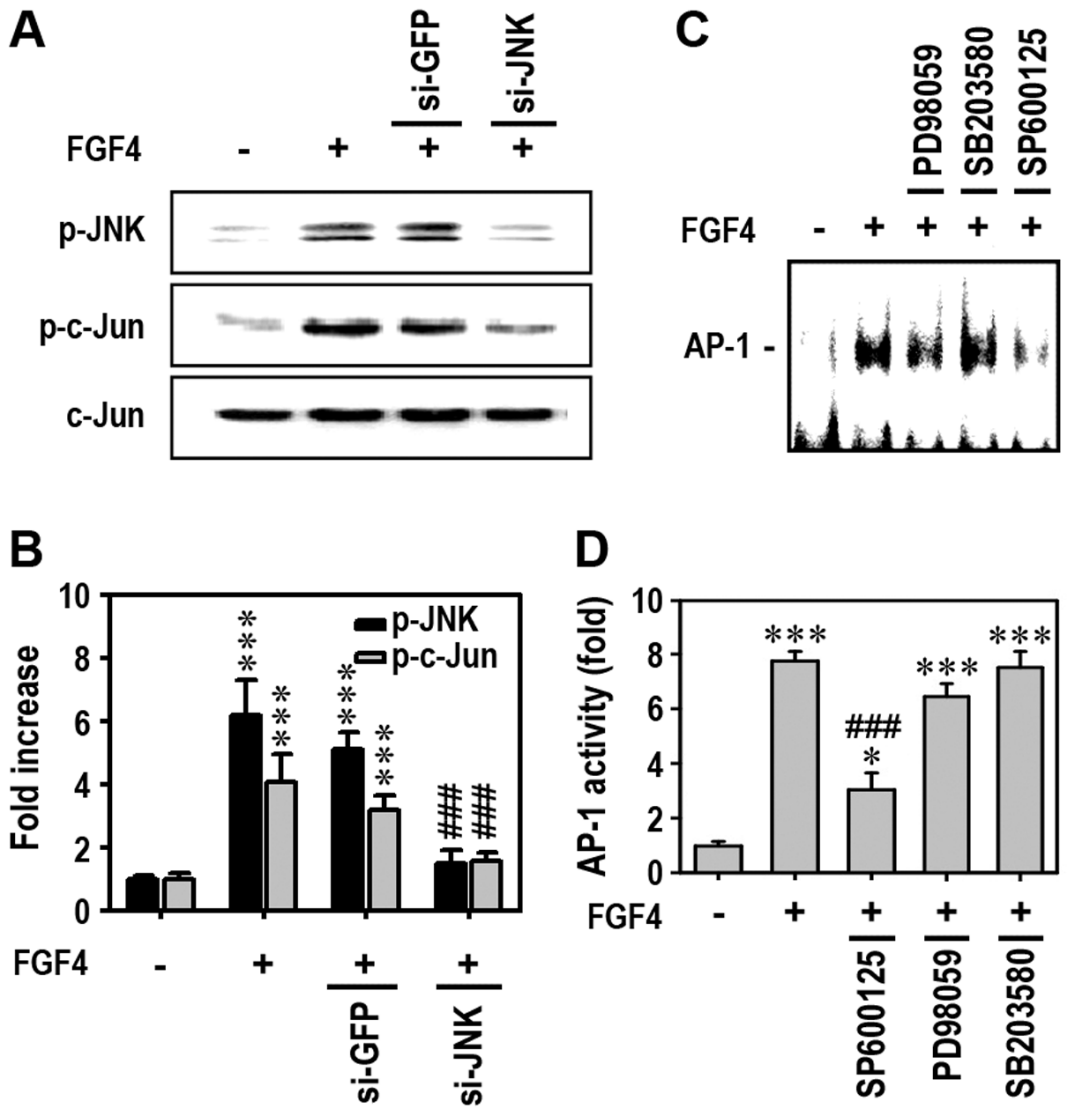
JNK acts as an upstream mediator of c-Jun phosphorylation and AP-1 activation. (A) mESCs transfected with siJNK were incubated in the presence or absence of 50 ng/ml FGF4 and then processed for Western blot analysis 1 h after treatment. (B) Data were quantified from triplicate experiments by densitometry after normalizing bands to total c-Jun protein. mESCs were also treated with each MAPK-specific inhibitor (10 µM) 30 min before addition of 50 ng/ml FGF4, and cells were adjusted to analyze AP-1-DNA binding activity by EMSA (C) or AP-1 activity using a luciferase assay kit (D) after 2 h of incubation. *p<0.05 and ***p<0.001 vs. untreated controls. ^###^p<0.001 vs. cells treated with 50 ng/ml FGF4 only.

Since c-Jun is known to play a role in transmitting proliferative signals in various cell types, we examined whether c-Jun acts as the downstream inducer of JNK signaling in FGF4-stimulated mESCs. Transfection with siJNK significantly reduced p-JNK and p-c-Jun levels in FGF4-stimulated cells, whereas it had no effect on total c-Jun protein (Figs. 5A and B). When p-JNK levels were reduced by treatment with SP600125, p-c-Jun levels in cells exposed to FGF4 were also clearly attenuated (data not shown). FGF4 treatment of cells increased AP-1 binding to its corresponding DNA, which was inhibited by addition of JNK inhibitor, but not ERK or p38 kinase inhibitor (Fig. 5C). Further, FGF4 treatment increased AP-1 transcriptional activity in the cells and this was significantly suppressed only by adding JNK inhibitor (Fig. 5D). JNK siRNA transfection showed similar results to the pharmacological inhibitor, SP600125, when AP-1 activity was determined (data not shown).

### FGF4 Prevents Osteoblastic Differentiation of mESCs

**Figure 6:**
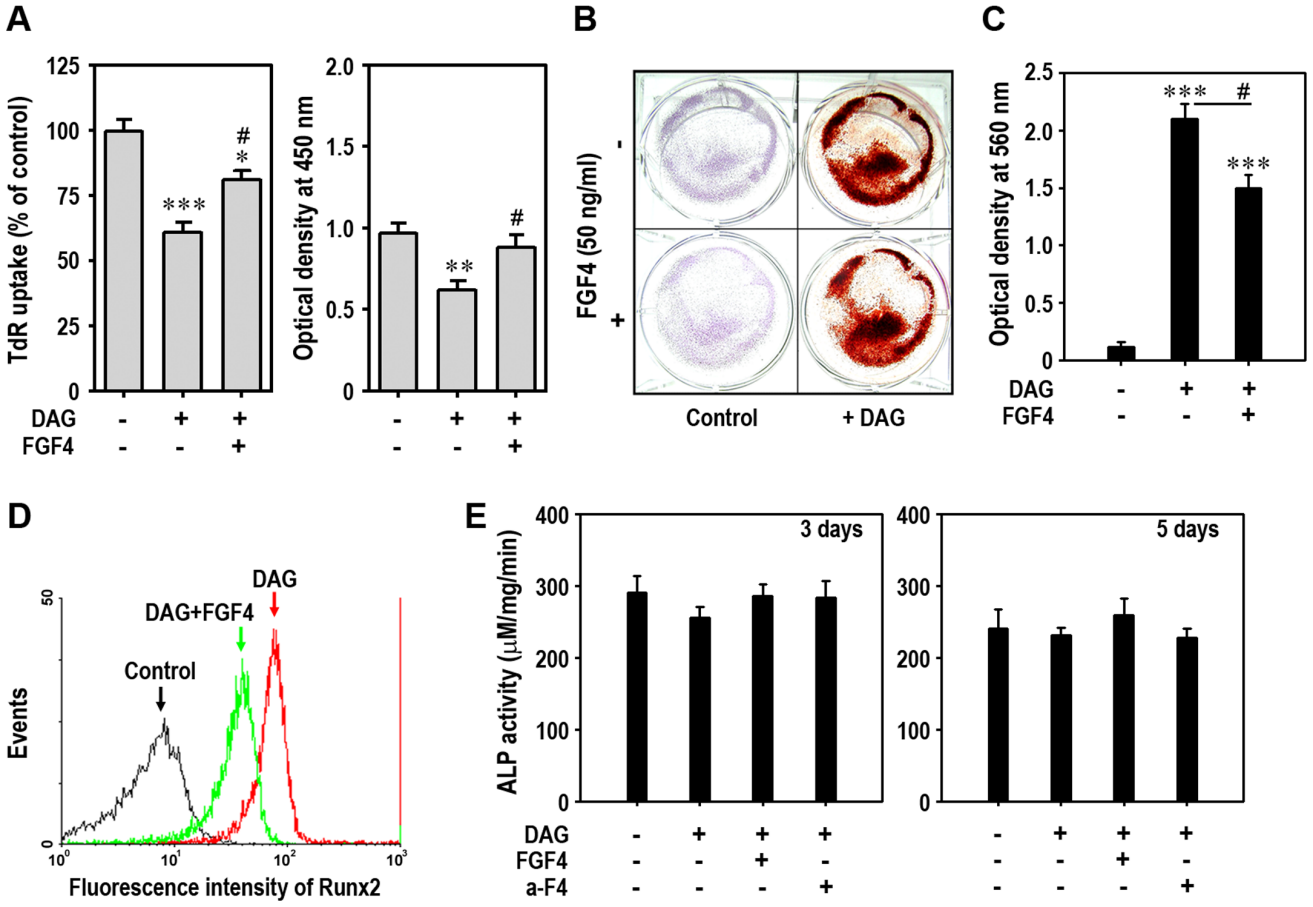
Exogenous FGF4 decreases osteogenic differentiation of mESCs. (A) mESCs were incubated in the presence of DAG and/or 50 ng/ml FGF4. After 5 days of incubation, DNA synthesis (left panel) and cell proliferation (right panel) rates were determined. At the same time, cells were processed for Alizarin red staining (B) and absorbance of the dye (C) was also determined at 560 nm. (D) Runx2 protein in mESCs cultured with DAG and/or 50 ng/ml FGF4 for 5 days was analyzed by flow cytometry. (E) ALP activity in the cells was measured after 3 and 5 days of differentiation. *p<0.05, **p<0.01 and ***p<0.001 vs. untreated control cells. ^#^p<0.05 vs. cells treated with DAG only.

We next investigated the effect of FGF4 on osteogenic differentiation of mESCs. When the cells incubated in DAG-supplemented medium for 5 days were processed for tritium uptake and cell proliferation assays, the levels of ^3^H-TdR incorporation were reduced to approximately 60% of untreated control cells (Fig. 6A, left panel). Cell proliferation assays also showed a DAG-mediated decrease in cell numbers and its inhibition by addition of exogenous FGF4 (Fig. 6A, right panel). Alizarin red-positive cells were clearly increased after DAG supplementation, whereas this increase was slightly reduced by adding 50 ng/ml FGF4 (Fig. 6B). Optical density also revealed a FGF4-mediated reduction in absorbance of the dye (Fig. 6C). Further, flow cytometric analysis shows that FGF4 reduces DAG-stimulated expression of Runx2 in mESCs (Fig. 6D). In contrast, ALP activity was not changed by DAG supplementation with or without FGF4 and/or anti-FGF4 antibody (Fig. 6E).

### FGF4 Suppresses mRNA Expression of Runx2 and Osterix in mESCs

**Figure 7:**
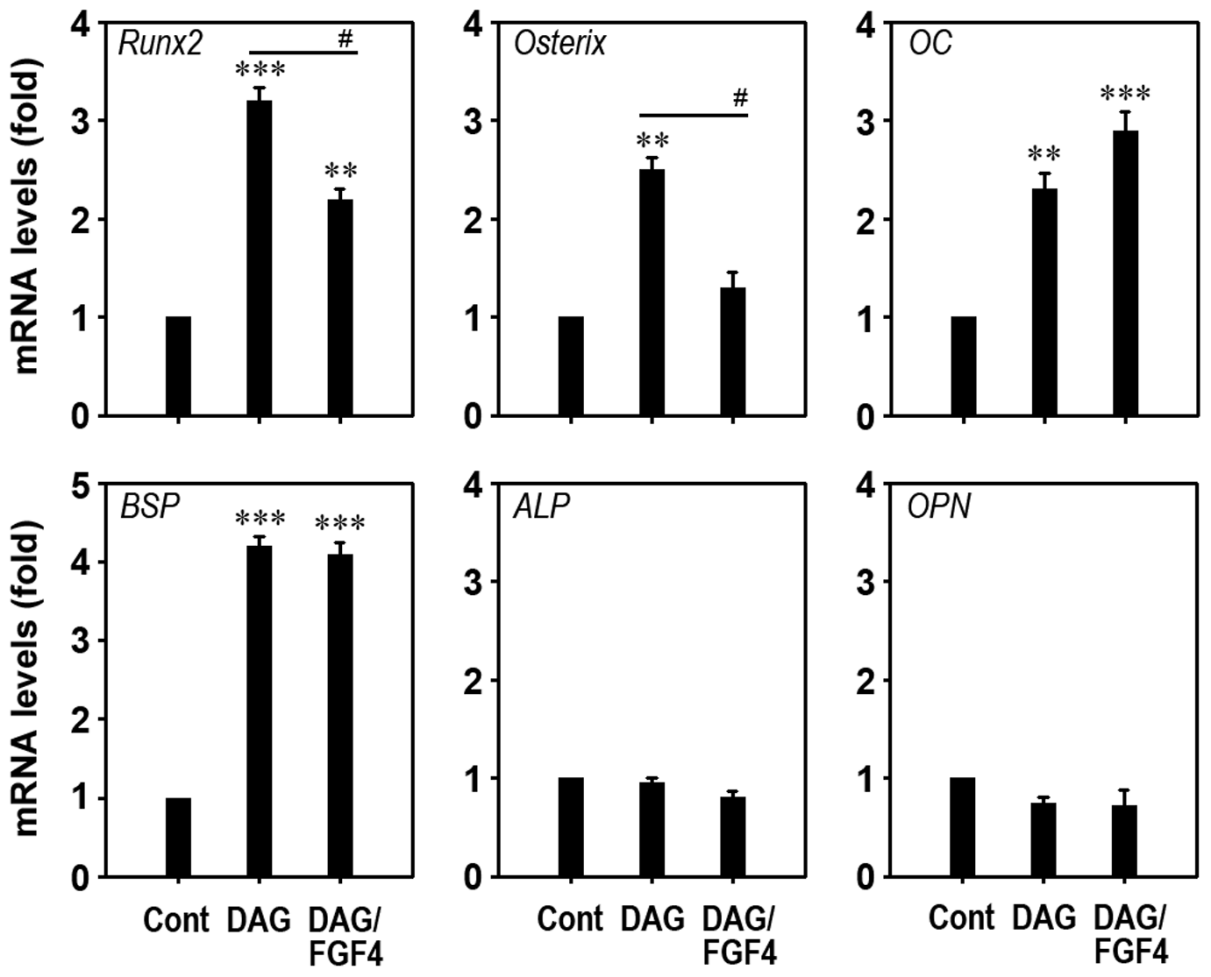
Addition of FGF4 diminishes DAG-induced expression of Runx2 and osterix in mESCs. Cells were incubated in the presence or absence of DAG and 50 ng/ml FGF4 for 24 h, and expression patterns of bone-specific genes were analyzed by real-time RT-PCR. **p<0.01 and ***p<0.001 vs. untreated control cells. ^#^p<0.05 vs. cells treated with DAG only.

Real time RT-PCR demonstrated significant increases in mRNA expression of Runx2, osterix, osteocalcin (OC), and bone sialoprotein (BSP), but not ALP or osteopontin (OPN) after 24 h of DAG supplementation (Fig. 7). DAG-mediated increases in mRNA expression of Runx2 and osterix were significantly attenuated by adding 50 ng/ml FGF4, whereas levels of OC and BSP were not changed under the same conditions. Moreover, mRNA levels specific to ALP and OPN were not changed regardless of the presence or absence of DAG and/or FGF4.

### FGF4 Stimulates Proliferation and Mineralization of hPDLSCs

**Figure 8:**
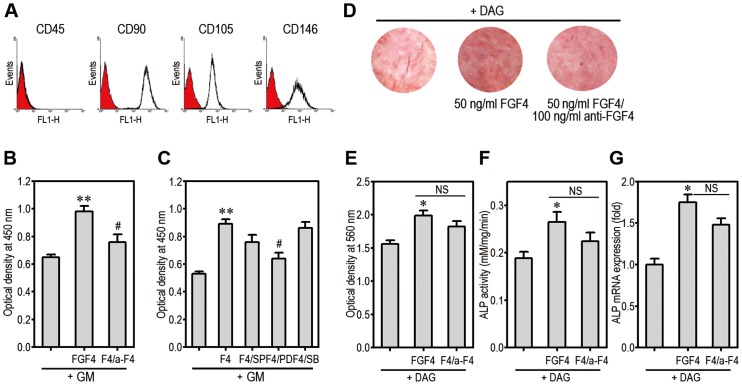
FGF4 stimulates proliferation and mineralization of hPDLSCs. (A) hPDLSCs at passage 3 were labeled with the indicated primary antibodies and then analyzed by flow cytometer. The cells were incubated for 48 h with 50 ng/ml FGF4 and/or 100 ng/ml (B), or with each of MAPK inhibitors (C) in 96-multiwell plates. The cells were processed for cell proliferation assay. **p<0.01 vs. GM alone. ^#^p<0.05 vs. FGF4 treatment alone. (D) hPDLSCs were incubated in the presence of DAG with and without 50 ng/ml FGF4 and 100 ng/ml anti-FGF4 for 14 days and then were processed for Alizarin red staining. (E) Absorbance of the dye was determined at 560 nm. hPDLSCs were also processed for analysis of ALP activity (F) and mRNA expression (G) after 5 days of differentiation. *p<0.05 vs. DAG alone. F4, FGF4; GM, growth medium; NC, non-significant.

Flow cytometric phenotyping of hPDLSCs showed that the cells were negative to CD45, whereas they expressed high levels of CD90 (99.9%), CD105 (97.5%), and CD146 (95.5%) (Fig. 8A). Exogenous addition of FGF4 (50 ng/ml) increased proliferation of the cells approximately to 1.5-fold and this increase was suppressed by adding anti-FGF4 (100 ng/ml) (Fig. 8B). Pretreatment with PD98059, but not with SP600125 or SB203580 prevented significantly FGF4-stimulated proliferation of the cells (Fig. 8C). The results from alizarin red staining revealed that FGF4 treatment augmented DAG-induced mineralization of hPDLSCs (Fig. 8D), while this was not reduced by anti-FGF4 antibody at a significant level. This was also supported by measuring optical density of the dye (Fig. 8E). The activity (Fig. 8F) and the expression (Fig. 8G) of ALP in DAG-treated hPDLSCs were stimulated by exogenous FGF4 addition, but its specific antibody did not affect such stimulation at a significant level.

### FGF4 also Stimulates Proliferation of mBMMSCs, but does not Affect Osteogenic Differentiation of the Cells

**Figure 9:**
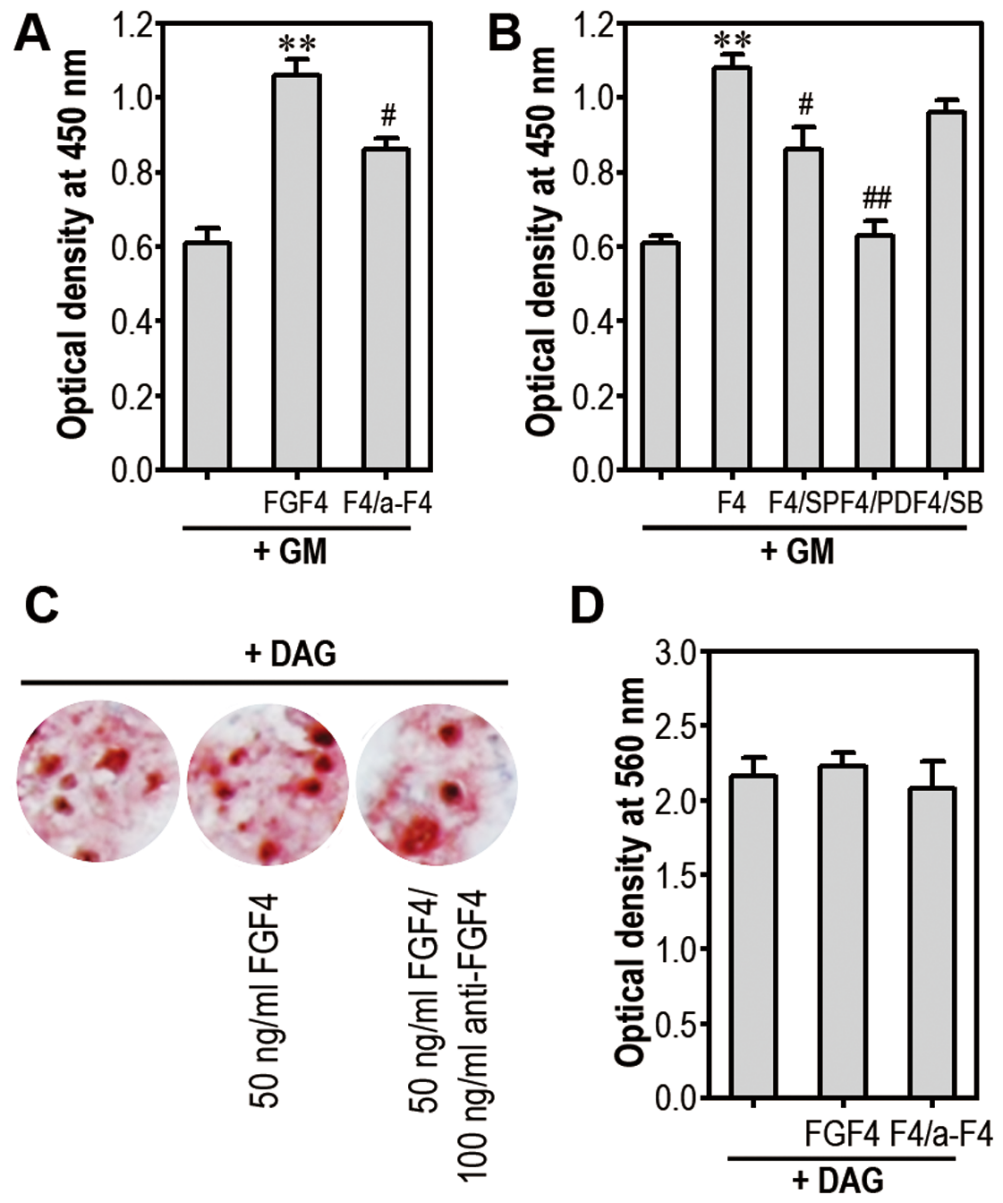
FGF4 stimulates proliferation of mBMMSCs via activation of MAPK. mBMMSCs were incubated with 50 ng/ml FGF4 and/or 100 ng/ml (A), or each of MAPK inhibitors (B) in 96-multiwell plates. After 48 h, these cells were processed for cell proliferation assay. **p<0.01 vs. GM alone. ^#^p<0.05 and ^##^p<0.01 vs. FGF4 treatment alone. (C) mBMMSCs were incubated in the presence of DAG with and without 50 ng/ml FGF4 and 100 ng/ml anti-FGF4 for 14 days and were then stained with Alizarin red. (D) The absorbance of the dye was measured at 560 nm.

The cells showed low level of CD106, but expressed high levels (approximately 50%) of CD29, CD44, and Sca-1 at passage 3 (data not shown). The cells more than 90% expressed Sca-1 after purifying using autoMACS system with biotin-conjugated anti-Sca-1 antibody and anti-biotin micro beads (data not shown). Exogenous addition of FGF4 (50 ng/ml) increased proliferation of mBMMSCs up to approximately 1.8-fold, compared to control (Fig. 9A). FGF4-mediated increase in proliferation was significantly suppressed by adding anti-FGF4 (100 ng/ml). The inhibitors specific to JNK and ERK, but not to p38 kinase, also inhibited FGF4-stimulated proliferation (Fig. 9B). Especially, treatment with PD98059 almost completely blocked cell proliferation. However, exogenous addition of FGF4 and/or anti-FGF4 to mBMMSCs did not affect the DAG-induced mineralization (Figs. 9C and 9D). mRNA expression of bone specific genes such as Runx2 and ALP in DAG-treated mBMMSCs was not changed by adding FGF4 and/or its neutralizing antibody (data not shown).

## Discussion

Numerous studies have reported that self-renewal, proliferation, migration, survival, and differentiation of stem cells are tightly modulated by growth factors [8], [25], [26]. Specifically, FGF4 is expressed in adult tissues as well as during embryonic development, and is involved in a variety of biological processes, including development, differentiation, cell proliferation, neurogenesis, tumorigenesis, and angiogenesis [6]. It has been demonstrated that FGF4 up-regulates neural progenitor cell proliferation and neuronal differentiation [7], survival and proliferation of trophoblast stem cells [9], [27], and hematopoietic potential of human long-term bone marrow cultures [28]. FGF4 also stimulates proliferation of Sca-1-positive BMMSCs [8]. In this study, FGF4 stimulated proliferation of mESCs as well as hPDLSCs and mBMMSCs. This finding is consistent with previous studies showing that several FGFs, such as FGF2, FGF4, and FGF7, stimulated proliferation of various types of cells including stem cells and fibroblasts [4], [16], [29], [30].

Growth factors, cytokines, and many types of stimuli activate JNK, ERK, and/or p38 kinase [31], [32]. FGFs also perform their biological functions by activating Ras-Raf-MAPK signaling cascades [17], [33]. Previous studies have reported that FGF4 stimulates proliferation of BMMSCs via activation of PI3K-Akt and ERK1/2 signaling pathways [8]. In addition to FGF4, phosphorylation of Akt and ERK1/2 was shown to be critical for FGF2-stimulated proliferation of BMMSCs as well as of neural progenitor cells [7], [8]. In parallel with these reports, considerable evidence highlights the importance of FGF-induced ERK1/2 signaling in the self-renewal of human multipotent adipose-derived stem cells [34]. FGFs also play important roles in chondrogenesis of embryonic limbs and early-stage frontonasal mesenchymal cell formation through MEK-ERK activation [35]. These findings suggest that ERK1/2 and Akt signaling pathways are predominantly involved in FGF-stimulated proliferation of various stem cells. However, in this study, FGF4 induced phosphorylation of JNK and ERK, but not p38, in mESCs, whereas only JNK activation was associated with FGF4-stimulated cell proliferation. Inconsistent with this result, FGF4-induced proliferation in hPDLSCs and mBMMSCs was significantly suppressed by inhibitor of ERK signaling. Further, the JNK-mediated signaling is also believed to be related in part to the FGF4-mediated increase of proliferation in mBMMSCs.

The present findings combined with previous reports suggest that FGF4 may stimulate proliferation of various types of cells, but in different ways. It is important to consider that the predominant signaling cascade activated by FGF4 may depend on the cell type being exposed to the factor [7]. Thus, cell type and origin could account for the diverse outcomes of FGF signaling on cell proliferation, survival, migration, and differentiation. It was reported that FGF2 induces phosphorylation of JNK, but not ERK or p38, in human MSCs [4]; however, this result is not a common observation. MAPKs are usually regulated coordinately; for example, mitogenic signals activate ERK and JNK, while stress signals simultaneously induce activation of JNK and p38. In the present study, FGF4 stimulated activation of both JNK and ERK in mESCs, although only the JNK signaling pathway was shown to be associated with FGF4-mediated proliferation of the cells. These results differ from a previous study showing that FGF4 increases proliferation of BMMSCs through ERK/Akt pathways [8]. It is likely that FGF4 activates ERK signaling in mESCs, but this activation affects other cellular events rather than proliferation. Consequently, we suggest that FGF4 facilitates proliferation through activation of MAPKs and this mechanism may be differed according to the origins of stem cells.

c-Jun is known as a proto-oncogene that transmits proliferative signals in various cell types [36]. JNK also enhances AP-1 activity by phosphorylating the active domain of c-Jun proteins [22]. Once MAPKs are activated, they affect AP-1 transcriptional activity. AP-1 is a family of homo- and/or hetero-dimers formed by Jun and Fos proteins [37], [38]. AP-1 regulates cellular proliferation, differentiation, and apoptosis via modulation of various downstream genes, and its role differs according to cell type and external or internal stimuli. It is believed that the main function of c-Jun is to transmit proliferative signals in cells [39], [40]. In this study, FGF4 was also found to induce phosphorylation of JNK in mESCs, which subsequently resulted in activation of c-Jun. In contrast, up-regulation of JunB was not observed in FGF4-treated moue ESCs (result not shown). It was reported that antagonistic activity between c-Jun and JunB controls cytokine-regulated mesenchymal-epidermal interactions in skin [40]. This suggests that the relative activation state of these AP-1 subunits plays an important role in determining the biological functions of AP-1 complexes in response to cytokines and growth factors. In this regard, it is suggested that c-Jun activation plays critical roles in cell proliferation stimulated by FGF4. Furthermore, this study demonstrated that JNK inhibitor, but not ERK or p38 inhibitor, prevents AP-1-DNA binding and transcriptional activity. Therefore, JNK-c-Jun signaling pathways might have key roles in FGF4-stimulated proliferation of mESCs. Additional experiments will be needed in order to clarify the mechanisms by which FGF4 stimulates proliferation in hPDLSCs and mBMMSCs.

We also investigated the effects of exogenous FGF on osteogenic differentiation of mESCs. FGF4 inhibited DAG-mediated reduction of ^3^H-TdR uptake levels and numbers of mESCs. It also attenuated DAG-induced osteoblastic differentiation as evidenced by the reduction of alizarin red-stained cells and of Runx2-specific fluorescence intensity. ALP activity was not changed either by treatment with FGF4 alone or in combination with its neutralizing antibody. It was reported that specific ALP activity remains relatively constant under maintenance conditions of mESCs, whereas addition of basic FGF reduces ALP activity associated with osteoblast activity during osteogenic differentiation [41]. In the present study, ALP activity at early stages of differentiation may not have been due entirely to induction of the osteogenic pathway. More detailed experiments will be needed in order to clarify the reason by which ALP activity is not increased in mESCs.

To better understand the effect of FGF4 on osteogenic differentiation, we analyzed the expression levels of bone-specific genes using real-time RT-PCR. The mRNA levels of Runx2, osterix, OC, and BSP were enhanced approximately 3.2-, 2.5-, 2.3-, and 4.2-fold, respectively, in the presence of DAG, compared to those of untreated control cells. Consistent with the enzymatic analysis of ALP, mRNA expression of ALP was not changed by supplementation with DAG and/or FGF4. Exogenous addition of FGF4 significantly diminished DAG-induced increases in the mRNA levels of Runx2 and osterix, but not of OC and BSP. Runx2 and osterix are essential osteoblast-specific transcription factors regulating bone differentiation [42], [43]. These factors activate a repertoire of genes during differentiation of pre-osteoblasts into mature osteoblasts and osteocytes [42], [44]. Osterix is also critical for differentiation of Runx2-expressing precursors into mature and functional osteoblasts, and expression of osterix mRNA and protein is positively regulated by Runx2 [43]. These findings suggest that FGF4 attenuates DAG-induced osteogenic differentiation by negatively regulating expression of these transcription factors in mESCs. OC is secreted mostly by mature osteoblasts during matrix calcification [45]; whereas BSP is known to regulate proliferation and differentiation of bone-forming cells [46]. Thus, it is likely that mature and functional osteoblasts express high levels of osteoblast markers including OC and BSP [43]. However, exogenous FGF4 addition did not affect DAG-mediated increases in OC and BSP expression in mESCs. A precise mechanism involved in this undesirable result could not be explained; however, it is important to consider that OC and BSP expression was not affected directly by Runx2 and/or osterix at early stages of osteogenic differentiation [45]. It has been proposed that increased OC and BSP expression in immature osteoblasts or pre-osteoblasts in the presence of DAG does not influence the process of mineralization at a significant level. It is considerable that osteoblastic markers, such as osteonectin and collagens, are also associated with osteogenic differentiation affected by FGF4 in the presence of DAG.

Our present findings revealed that exogenous FGF4 augmented mineralization in hPDLSCs with the attendant increases in ALP activity and expression. In contrast, co-treatment with its specific antibody did not inhibit the FGF4-stimulated osteogenic differentiation in the cells and also did not reduce the ALP activity and expression at a significant level. In this regard, it is assumed that FGF4 acted as an indirect stimulator of mineralization in hPDLSCs. It is also postulated that the population of these cells did not have a strong potential as mesenchymal stem cells. However, mBMMSCs did not show any changes in mineralization after treatment with FGF4 and/or anti-FGF4. Although this result indicates a different response of stem cells to FGF4 according to the origins, further experiments are required for the elucidation of mechanisms involved in the different responses to FGF4.

In summary, we demonstrated that FGF4 induced activation of JNK and c-Jun and eventually AP-1, which was closely associated with FGF4-induced proliferation of mESCs. In contrast, FGF4 suppressed DAG-induced osteogenic differentiation by down-regulating Runx2 and osterix. FGF4 also facilitated proliferation of hPDLSCs and mBMMSCs through the activation of JNK and/or ERK-mediated signaling pathways. In addition, FGF4 has different effects on osteogenic differentiation of these mesenchymal stem cells; FGF4 stimulated mineralization of hPDLSCs, but not of mBMMSCs, whereas anti-FGF4 did not affect DAG-mediated mineralization in both cells.
